# Non-small cell lung cancer with synchronous brain metastases: Identification of prognostic factors in a retrospective multicenter study (HOT 1701)

**DOI:** 10.1093/noajnl/vdae168

**Published:** 2024-10-05

**Authors:** Yoshihito Ohhara, Tetsuya Kojima, Osamu Honjo, Noriyuki Yamada, Toshitaka Sato, Hirofumi Takahashi, Kei Takamura, Taichi Takashina, Noriaki Sukoh, Hisashi Tanaka, Yasutaka Kawai, Yuka Fujita, Keiki Yokoo, Fumihiro Hommura, Toshiyuki Harada, Ryoichi Honda, Toraji Amano, Hirotoshi Dosaka-Akita, Satoshi Oizumi, Ichiro Kinoshita

**Affiliations:** Department of Medical Oncology, Hokkaido University Hospital, Kita-ku, Japan; Department of Respiratory Medicine, KKR Sapporo Medical Center, Toyohira-ku, Japan; Department of Respiratory Medicine, Sapporo Minamisanjo Hospital, Chuo-ku, Japan; Department of Respiratory Medicine, National Hospital Organization Hokkaido Cancer Center, Kikusui, Japan; Department of Respiratory Medicine, KKR Sapporo Medical Center, Toyohira-ku, Japan; Department of Respiratory Medicine, Hokkaido University Hospital, Kita-ku, Japan; Department of Respiratory Medicine, Obihiro Kosei Hospital, Obihiro, Japan; Department of Respiratory Medicine, Iwamizawa Municipal General Hospital, Iwamizawa, Japan; Department of Respiratory Medicine, National Hospital Organization Hokkaido Medical Center, Nishi-ku, Japan; Department of Respiratory Medicine, Hirosaki University Graduate School of Medicine, Hirosaki city, Japan; Department of Respiratory Medicine, Oji General Hospital, Tomakomai, Japan; Department of Respiratory Medicine, National Hospital Organization Asahikawa Medical Center, Asahikawa, Japan; Department of Respiratory Medicine, Teine Keijinkai Hospital, Teine-ku, Japan; Department of Respiratory Medicine, Sapporo City General HospitalChuo-ku, Japan; Center for Respiratory Diseases, Japan Community Healthcare Organization Hokkaido Hospital, Toyohira-ku, Japan; Department of Respiratory Medicine, Asahi General Hospital, Chiba Prefecture, Japan; Clinical Research and Medical Innovation Center, Hokkaido University Hospital, Kita-ku, Japan; Department of Medical Oncology, Hokkaido University Hospital, Kita-ku, Japan; Department of Respiratory Medicine, National Hospital Organization Hokkaido Cancer Center, Kikusui, Japan; Department of Medical Oncology, Hokkaido University Hospital, Kita-ku, Japan

**Keywords:** brain metastasis, non-small cell lung cancer, prognostic factor

## Abstract

**Background:**

Non-small-cell lung cancer (NSCLC) is associated with a high incidence of brain metastasis (BM), and the prognosis of patients with NSCLC and BM is poor. This study aimed to identify the prognostic factors and elucidate the survival rates of Japanese patients with NSCLC and BM at initial diagnosis.

**Methods:**

HOT 1701 is a retrospective multicenter study of patients with NSCLC and BM at initial diagnosis. The medical records of all consecutive patients diagnosed with advanced or recurrent NSCLC and BM at 14 institutions of the Hokkaido Lung Cancer Clinical Study Group Trial (HOT) in Japan were reviewed. The participants were categorized based on the presence or absence of driver mutations. The Kaplan–Meier method was used to estimate median overall survival (OS). Univariate and multivariate analyses were performed to identify prognostic factors in these patients.

**Results:**

Among 566 patients with NSCLC and BM, the median OS was 11.8 months. Patients with driver mutations survived longer than those without driver mutations. The univariate and multivariate analyses revealed 6 independent prognostic factors: age ≥65 years, poor performance status, T factor, absence of driver gene mutations, presence of extracranial metastases, and number of BM. According to the prognostic score based on these 6 factors, the patients were stratified into 3 risk groups: low-, intermediate-, and high-risk, with median OS of 27.8, 12.2, and 2.8 months, respectively.

**Conclusions:**

We developed a new prognostic model for patients with NSCLC and BM, which may help determine prognosis at diagnosis.

## Lay Summary

Non-small-cell lung cancer (NSCLC) is a very aggressive type of lung cancer that often spreads to the brain. The authors of this study wanted to better understand which factors determine how long a patient lives once NSLC metastasizes to the brain. To do this, the authors reviewed the medical records of 566 patients in Japan with metastases in their brains from NSCLC. Their results showed that, on average, patients lived for 11.8 months. They identified 6 factors that affected how long they lived. These factors included age, overall health status, the severity of the original lung cancer, genetic differences in the cancer, whether the cancer had spread to other parts of the body, and the total number of different metastases in the brain. Based on these factors, the authors identified 3 groups of patients, with those in the high-risk group having much shorter survival compared to those in the low-risk group.

**Key Points**

- Six independent prognostic factors were identified for patients with NSCLC and BM.
- Patients can be at low-, intermediate-, or high-risk based on the prognostic score.
- Treatment approach can be determined based on the prognostic score.

**Importance of the Study**

Brain metastasis (BM) frequently occurs as a complication of non-small-cell lung cancer (NSCLC), with a total incidence of 40% during disease progression and a prevalence of 15%–20% at initial diagnosis. The presence of BM is of considerable clinical significance in patients with NSCLC because it is associated with unfavorable prognosis, extremely low survival rates, and deterioration of the quality of life. Detecting BM prior to surgery can potentially alter the treatment approach for individuals who would otherwise be categorized as having an early-stage disease. Univariate and multivariate analyses revealed 6 independent prognostic factors: age ≥65 years, poor performance status, T factor, absence of driver gene mutations, presence of extracranial metastases, and number of BM. Stratification of patients according to risk based on these prognostic factors may help determine the prognosis of patients at initial diagnosis, leading to appropriate treatment.

Non-small-cell lung cancer (NSCLC), one of the most lethal cancers, accounts for a substantial proportion of cancer-related mortality worldwide. Despite advances in diagnostic techniques, therapeutic interventions, and patient care, NSCLC presents formidable challenges, one of which is the occurrence of brain metastasis (BM), which considerably alters the prognosis and treatment landscape of the affected individuals. BM frequently occurs as a complication of NSCLC, with a total incidence of 40% during disease progression and a prevalence of 15%–20% at initial diagnosis.^1,2^ The presence of BM is of considerable clinical significance in patients with NSCLC because it is associated with unfavorable prognosis, resulting in extremely low survival rates (5–18.9 months)^3^ and deterioration of the quality of life.^3^

Currently, the criteria for conducting brain imaging in patients newly diagnosed with NSCLC are primarily determined by clinical stage. According to the National Comprehensive Cancer Network, brain magnetic resonance imaging (MRI) is recommended for patients with stages II–IV disease and is optional for those with stage IB disease.^4^ In contrast, the eighth edition of the American Joint Committee on Cancer restricts brain imaging to clinical stages III and IV.^5^ However, owing to variations in these 2 sets of guidelines and the occurrence of BM even in the early stages of NSCLC, a more robust risk prediction approach that does not rely solely on the clinical stage should be implemented.^6,7^

Only a limited number of studies have identified predictors of BM in NSCLC. Mujoomdar et al. found correlations between primary tumor size, cell type, lymph node stage, and the likelihood of BM in patients with NSCLC.^8^ Their findings suggested that larger tumors, adenocarcinoma, undifferentiated NSCLC, and advanced lymph node stage are associated with increased risk of BM. However, their study did not provide prediction models based on these factors, rendering the direct application of their results in clinical settings difficult. Furthermore, their study lacked in-depth analyses of the computed tomography (CT) characteristics of primary tumors. Detecting BM prior to surgery can potentially alter the treatment approach for individuals who would otherwise be categorized as having an early-stage disease.

Recent investigations have identified specific driver genes as noteworthy predictors of BM.^9–11^ Notably, genes encoding epidermal growth factor receptor (*EGFR*) and anaplastic lymphoma kinase (*ALK*) have been linked to the distinct CT features of primary tumors.^12,13^

EGFR tyrosine kinase inhibitors (EGFR-TKIs) considerably improve the survival rate of patients with advanced NSCLC harboring *EGFR* mutations.^14–16^ However, in a previous study, patients with *EGFR*-mutated NSCLC, who also developed BM, experienced a comparatively shorter median overall survival (OS, 25.1 months) than those without BM (30.2 months).^17^ Studies have indicated that patients with NSCLC harboring *EGFR* mutations are at a higher risk of developing BM than those with *EGFR* wild-type tumors.^18–20^ Consequently, the prevention of metachronous BM is critical for improving the survival rate of patients with advanced *EGFR*-mutated NSCLC.

Prognostic factors for BM in Japanese patients with NSCLC have not yet been investigated. The aim of this study was to stratify risk groups according to prognostic factors in Japanese patients with NSCLC who had BM at diagnosis and elucidate their OS.

## Materials and Methods

### Study Design and Patients

This was a retrospective multicenter study (HOT 1701) of patients with NSCLC and BM at initial diagnosis. It was conducted at 14 institutions between January 2008 and December 2014 after approval by the institutional review boards of all institutions. The 14 institutions belonged to the Hokkaido Lung Cancer Clinical Study Group Trial (HOT) in Japan, namely, Hokkaido University Hospital (Sapporo), KKR Sapporo Medical Center (Sapporo), Sapporo Minamisanjo Hospital (Sapporo), National Hospital Organization Hokkaido Cancer Center (Sapporo), Obihiro Kosei Hospital (Obohiro, Hokkaido), Iwamizawa Municipal General Hospital (Iwamizawa, Hokkaido), National Hospital Organization Hokkaido Medical Center (Sapporo), Hirosaki University Hospital (Hirosaki, Aomori Prefecture), Oji General Hospital (Tomakomai, Hokkaido), National Hospital Organization Asahikawa Medical Center (Asahikawa, Hokkaido), Teine Keijinkai Hospital (Sapporo), Sapporo City General Hospital (Sapporo), Japan Community Healthcare Organization Hokkaido Hospital (Sapporo), and Asahi General Hospital (Asahi, Chiba Prefecture). The medical records of all consecutive patients diagnosed with advanced or recurrent NSCLC with BM at initial diagnosis were reviewed. We used the Union for International Cancer Control TNM classification of malignant tumors for classifying tumors. The inclusion criteria for the participants were as follows: diagnosis of NSCLC using cytological or histological methods and BM that could be evaluated using contrast-enhanced CT or MRI at the time of diagnosis or postoperative recurrence. The exclusion criteria included cases in which information regarding the patient and prognosis could not be obtained from medical records, and patients with BM recurrence after chemoradiotherapy (as it was unclear whether or not they were cured by chemoradiotherapy).

### Ethics Approval

This study was registered at the UMIN-CTR (registration number: UMIN000030313, date of first registration: December 8, 2017). The institutional review board of Hokkaido University Hospital provided ethics approval for this study (approval number: 017-0352) and waived the requirement of obtaining informed consent from the study participants because anonymized data were analyzed. The study was conducted in accordance with the principles of the Declaration of Helsinki.

### Categorization of Participants

The participants were categorized based on the presence or absence of driver mutations. *EGFR* mutations were detected using real-time polymerase chain reaction (PCR) or the peptide nucleic acid-locked nucleic acid PCR clamp method. *ALK* fusion was detected using fluorescent in situ hybridization, highly sensitive immunohistochemical staining, or real-time PCR.

### Determination of OS

The final follow-up was performed in December 2018. The Kaplan–Meier method was used to estimate the median OS, and differences were determined using the log-rank test. OS was defined as the period between the date of BM diagnosis and the date of death or last follow-up, whichever occurred earlier.

### Identification of Prognostic Factors

Univariate and multivariate analyses were performed to identify the prognostic factors in patients with NSCLC and BM. The following variables were considered in the multivariate analyses: age, smoking status, Eastern Cooperative Oncology Group performance status (PS), T factor, N factor, extracranial metastases, driver mutation (*EGFR* and *ALK*), and number of BM. Staging systems used in this study are presented in Supplementary Table 1. Multiplicity analysis was not performed in this study. Factors associated with mortality risk were examined using univariate analysis (chi-square test). Variables with *P* < .05, determined using univariate analysis, were incorporated in the subsequent multivariate analysis with the Cox proportional hazards model. Statistical significance was defined at *P* < .05. All statistical analyses were performed using SPSS version 26.0 (IBM Corp.).

## Results

### Patients’ Characteristics

In total, 566 patients with NSCLC and BM at initial diagnosis were enrolled in the HOT 1701 study. The flowchart describing the number of patients identified and then excluded is presented in Supplementary Figure 1. The patient characteristics are shown in Table 1. BMs were detected using CT in 14 patients and using MRI in 552 patients. Among the 566 patients with NSCLC and BM, 441 (78%) received chemotherapy and 415 (73%) received local treatment for BM (whole-brain radiotherapy, 270 (48%); stereotactic radiotherapy or stereotactic radiosurgery, 171 (30%); and surgery, 60 (11%)). All cases with BM, including those with meningeal dissemination, were included. The patients were identified as having meningeal dissemination based on the MRI report. Cerebrospinal fluid testing was not required. In total, 38 of the 566 cases (7%) were complicated with meningeal dissemination.

**Table 1.** Characteristics of the Patients

		*N* = 566	Percentage
**Age** (years), median (range)		66	(24–89)
**Sex**			
	Male	350	62%
	Female	216	38%
**ECOG PS**			
	0–1	374	66%
	2	125	22%
	3–4	67	11%
**Smoking habit**			
	Never	153	27%
	Former or current	405	72%
	Unknown	8	1%
**Stage**			
	Stage IV	539	95%
	Recurrence	27	5%
**Histological type**			
	Adenocarcinoma	431	76%
	Squamous cell carcinoma	67	12%
	Others	68	12%
**T factor**			
	T1	90	16%
	T2	183	32%
	T3	96	17%
	T4	190	34%
	Tx	7	1%
**N factor**			
	N0	98	17%
	N1	49	9%
	N2	168	30%
	N3	250	44%
	Nx	1	0%
**Extracranial metastases**			
	Absent	156	28%
	Present	410	72%
**Driver gene mutation** (*EGFR/ALK*)		176	31%
**Number of BM**			
	1	182	32%
	2–3	135	24%
	≥4	249	44%
**Symptoms of BM**			
	Absent	331	58%
	Present	234	41%
	Unknown	1	0%
**Brain edema**			
	Absent	221	39%
	Present	341	60%
	Unknown	4	1%
**Meningitis**			
	Absent	496	88%
	Present	38	7%
	Unknown	32	6%
**Treatment**			
	Local treatment for BM^a^	415	73%
	Whole-brain radiotherapy (WBRT)	270	48%
	Stereotactic radiosurgery or stereotactic radiotherapy (SRS)	171	30%
	Surgery	60	11%
	Chemotherapy^b^	441	78%
	WBRT + SRS + Surgery	4	0.7%
	WBRT + SRS	34	6%
	WBRT + Surgery	29	5.1%
	SRS + Surgery	15	2.6%
	WBRT only	203	35.9%
	SRS only	118	20.8%
	Surgery only	12	2.1%

ECOG, Eastern Cooperative Oncology Group; PS, performance status; EGFR, epidermal growth factor receptor; ALK, anaplastic lymphoma kinase; BM, brain metastases.

^a^Includes all treatments for BM during patients’ clinical course.

^b^Patients treated with EGFR or ALK-TKI are included. In total, 441 patients were administered cytotoxic chemotherapy, including platinum-doublet, taxane, EGFR-TKI (203 patients), and ALK-TKI (7 patients).

### Overall Survival

**Figure 1.**
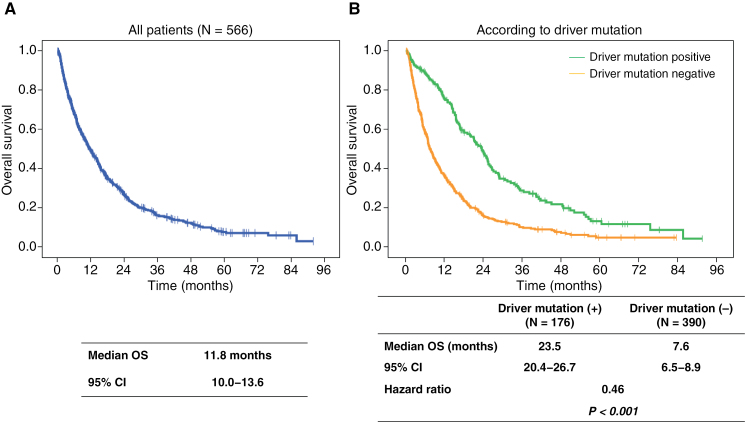
Overall survival (A) of all patients and (B) according to the presence or absence of driver mutations. OS, overall survival; CI, confidence interval.

Median OS was 11.8 (95% confidence interval [CI], 10.0–13.6) months on the cutoff date (Figure 1A). Patients with driver mutations (*N* = 176) survived longer than those without any driver mutation (*N* = 390; median OS, 23.5 vs. 7.6 months, *P* < .001; Figure 1B). Furthermore, patients who received local treatment and chemotherapy for BM had significantly longer survival (OS [median], 16.6 months; 95% CI: 14.7–18.5 and OS [median], 15.2 months; 95% CI: 12.6–17.8, respectively) than those who did not (OS [median], 8.3 months; 95% CI: 6.1–10.6 [*P* < .001] and OS [median]; 2.9 months, 95% CI: 2.3–3.5; *P* < .001), respectively. We also compared patients with adenocarcinoma and those with squamous cell carcinoma (Supplementary Table 2). The OS in patients with adenocarcinoma (OS [median], 14.2 months; 95% CI: 12.4–16.0) was significantly longer than in those with squamous cell carcinoma (OS [median], 7.3 months; 95% CI: 6.0–8.7, *P* = .006 [log-rank test]).

### Prognostic Factors

The univariate (Table 2) and multivariate (Table 3) analyses revealed the following 6 independent prognostic factors: age ≥65 years (*P* = .002), poor PS (*P* < .001), T factor (*P* < .001), absence of driver gene mutations (*P* < .001), presence of extracranial metastases (ECM; *P* < .001), and number of BM (*P* < .001).

**Table 2.** Univariate Analysis for Overall Survival

	Hazard ratio	95% confidence interval	*P* value
Sex, male vs. female	0.603	0.496–0.733	<.001
Age, <65 years vs. ≥65 years	1.474	1.22–1.782	<.001
Smoking habit, never vs. other	1.346	1.09–1.661	.006
Stage IV vs. recurrence	0.65	0.406–1.043	.074
ECOG PS, 0–1 vs. 2–4	3.204	2.624–3.912	<.001
T factor, T1–2 vs. T3–4	1.404	1.165–1.693	<.001
N factor, N0 vs. N1–3	1.732	1.34–2.238	<.001
Extracranial metastases	1.656	1.336–2.051	<.001
Driver mutation (EGFR, ALK), no vs. yes	0.456	0.371–0.562	<.001
Number of BM, 1–3 vs. ≥4	1.162	1.058–1.277	.002
Symptoms of BM, absent vs. present	1.308	1.083–1.579	.005
Meningitis, absent vs. present	1.387	0.971–1.98	.072

ECOG, Eastern Cooperative Oncology Group; PS, performance status; EGFR, epidermal growth factor receptor; ALK, anaplastic lymphoma kinase; BM, brain metastases.

**Table 3.** Multivariate Analysis for Overall Survival

	Hazard ratio	95% confidence interval	*P* value
Age, <65 years vs. ≥65 years	1.353	1.115–1.642	.002
Smoking habit, never vs. other	0.894	0.684–1.168	.41
ECOG PS, 0–1 vs. 2–4	3.034	2.46–3.743	<.001
T factor, T1–2 vs. T3–4	1.538	1.26–1.877	<.001
N factor, N0 vs. N1–3	1.216	0.925–1.597	.161
Extracranial metastases	1.748	1.372–2.229	<.001
Driver mutation (*EGFR, ALK*), no vs. yes^a^	0.4	0.318–0.504	<.001
Number of BM, 1–3 vs. ≥4^b^	1.225	1.108–1.355	<.001

ECOG, Eastern Cooperative Oncology Group; PS, performance status; EGFR, epidermal growth factor receptor; ALK, anaplastic lymphoma kinase; BM, brain metastases.

^a^Sex was excluded from the multivariate analysis as it correlated with driver mutations.

^b^Symptoms of BM were excluded in multivariate analysis as they correlated with the number of BM.

### Stratification Into Risk Groups

Patients with NSCLC and BM were categorized into 3 groups based on the prognostic score established using the 6 factors (Table 4). The hazard ratio of poor PS and absence of driver mutation was large; therefore, we scored it as follows: PS, 0–1 = 0 points, 2 = 1 point, and 3–4 = 2 points; the presence of driver mutation = 0 points and absence = 2 points. Moreover, other risk factors, such as age, T factor, presence of ECM, and number of BM, were scored as 0 or 1 point, as described in Table 4. We categorized the patients into 3 risk groups based on the total points as follows: low-risk, 0–2 points; intermediate-risk, 3–5 points; and high-risk, 6–8 points.

**Table 4.** Risk Groups According to Prognostic Factors

Score	0	1	2
Age	<65 years	≥65 years	
ECOG PS	0, 1	2	3, 4
T factor	1, 2	3, 4	
Extracranial metastases	Absent	Present	
Number of brain metastases	1–3	≥4	
Driver Mutation	Yes		No
	Prognostic factors score		
Low-risk	0–2		
Intermediate-risk	3–5		
High-risk	6–8		

ECOG, Eastern Cooperative Oncology Group; PS, performance status.

**Figure 2.**
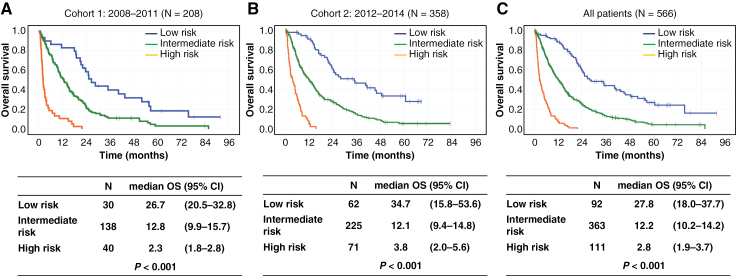
Overall survival according to risk groups. (A) Cohort 1: 2008–2011 (*N* = 208), (B) Cohort 2: 2012–2014 (*N* = 358), (C) All patients (*N* = 566). OS, overall survival; CI, confidence interval.

We analyzed the OS of patients based on risk groups. We initially analyzed an exploratory cohort of 208 patients between 2008 and 2011 (Figure 2A). We analyzed 358 patients between 2012 and 2015 as the validation cohort (Figure 2B). The OS of patients in the high-, intermediate-, and low-risk groups was 2.8 (*N* = 111; 95% CI: 1.9–3.7), 12.2 (*N* = 363; 95% CI: 10.2–14.2), and 27.8 (*N* = 92; 95% CI: 18.0–37.7) months, respectively (Figure 2C).

### Comparison With Graded Prognostic Assessment Score

We used our data to analyze graded prognostic assessment (GPA) scores. The GPA prognostic prediction model was also validated in our study cohort. The data are presented in Supplementary Figure 2.

## Discussion

The increasing occurrence of BM in patients with NSCLC and poor prognosis prompted us to investigate the prognostic factors for patients with NSCLC and BM at diagnosis, especially in Japanese patients. To the best of our knowledge, this is the first study to develop a prognostic model for Japanese patients who had BM at diagnosis. Despite substantial improvements in NSCLC care, the prognosis of patients with BM remains poor.

Targeted medications used to treat BM in patients with NSCLC are limited by the blood–brain barrier, which prevents the drugs from reaching the brain parenchyma, thereby decreasing their efficacy.^21^ The emergence of resistance to drugs, particularly osimertinib, is another critical issue, due to which fourth-generation tyrosine kinase inhibitors have not yet been developed.^22^ Additionally, the median survival time of patients is typically 3–8 months, leaving little time for treatment. Therefore, considering the current situation, additional mechanism-related research should be performed urgently to identify prognostic markers for patients with NSCLC and BM.

Six prognostic factors were determined in this study, based on which the patients were divided into low-, intermediate-, and high-risk groups. Patients in the high-risk group showed poorer survival rates than those in the intermediate- and low-risk groups. The GPA prognostic prediction model was also validated in our study cohort. The similarity between the prognostic factor score in this study and GPA is that 4 items (age, PS, extracerebral metastasis, number of BM) are included as prognostic factors. The difference is that in this study, T stage, N stage, and driver gene mutations, which are important in the clinical practice of lung cancer, were included, and we believe that our prognostic prediction score is more in line with actual clinical practice.

Supplementary Figure 2 shows the reproducibility of the GPA score in the cases studied in this study, and the new prognostic prediction score developed in this study may be universally applicable to patients with lung cancer and BM.

The prognosis of patients with advanced NSCLC carrying *EGFR* mutations has improved significantly over the past 20 years owing to the development of EGFR-TKIs. The median OS in clinical trials involving first- or second-generation EGFR-TKIs has been 19.3–33.2 months.^23,24^ Lee et al. found that among 166 patients who derived clinical benefits from the use of first-generation EGFR-TKIs (gefitinib and erlotinib), 26% experienced central nervous system failure.^25^ Additionally, *EGFR*-mutant patients with BM survived longer^18^ and showed a higher incidence of metachronous BM than patients with NSCLC harboring wild-type *EGFR*.^26^ However, because of insufficient data, the authors indicated that patients with *EGFR*-mutant NSCLC who have metachronous BM should receive greater attention.

Zhu et al.^27^ investigated the epidemiology of synchronous BM in patients with NSCLC. They used logistic regression and Cox regression to identify factors associated with synchronous BM incidence and cancer-specific survival. They reported a 12.58% incidence of synchronous BM in patients with NSCLC and the median cancer-specific survival duration of 5 months. Patients with younger age, female sex, and adenocarcinoma had higher odd ratios for developing synchronous BM.

In our study, we focused on patients with synchronous BM at initial diagnosis and stratified the patients according to the risk groups based on the prognostic factors. The new prognostic model based on the 6 prognostic factors in patients with NSCLC and BM may help determine prognosis at diagnosis.

Our study has several limitations. First, as this was a multicenter study, all patients did not receive similar treatments. Furthermore, imaging tests were not performed in a unified manner at multiple facilities. Second, owing to the retrospective design of the study, a selection bias was inevitable. Additionally, the exclusion of patients with BM recurrence after chemoradiotherapy might have led to selection bias in the study. Third, we did not perform any multiplicity analysis. Fourth, although we developed and evaluated the prognostic score in the internal validation cohort, external validation in different cohorts is required to generate the most reliable results. Analysis within the same cohort can provide limited helpful information, and more information is needed to fully demonstrate the scores’ generality. Fifth, driver gene mutations were not examined in all cases. *EGFR* mutations were not examined in 161 cases, and the *ALK* fusion gene was not examined in 395 cases. *ALK* fusion gene testing was approved for insurance coverage in Japan in 2012; therefore, many cases remained untested. There were also cases where genetic testing could not be performed due to insufficient sample volume. Sixth, we did not investigate driver gene mutations other than *EGFR* and *ALK*. In Japan, *ROS1* fusion gene testing has been covered by insurance since 2017, and *BRAF V600E* mutation testing since 2018, but they were hardly investigated in lung cancer cases from 2008 to 2014 when our patients were enrolled. Furthermore, *PD-L1* expression is associated with increased tumor proliferation, aggressiveness, and reduced patient survival in NSCLC. Higher malignancy grades and lymph node status are associated with elevated *PD-L1* expression. Patients with low *PD-L1* expression in adenocarcinoma had longer OS than those with high expression.^28,29^ ROS1 fusion genes, present in various tumors, promote cell proliferation, activation, and cell cycle progression by activating downstream signaling pathways, accelerating NSCLC development and progression. ROS1 inhibitors are effective in patients with ROS1-positive NSCLC and are used as first-line treatment.^30^ BRAFV600E mutation, found in 1%–2% of lung adenocarcinomas, acts as an oncogenic driver. As our study was performed using data from 2008 to 2014, testing of these driver mutations was not a common practice then and as specified, testing of these mutations were covered in insurance from 2017 and 2018 onward. Seventh, the patients were enrolled over a long duration (2008–2014), wherein different patients had different levels of access to brain imaging and treatments. We conducted an analysis comparing data between 2008–2010 and 2011–2014 to clarify the differences (Supplementary Table 3). No significant differences were observed in patient background between Cohort 1 (2008–2011) and Cohort 2 (2012–2014) except for smoking habits. Patients in Cohort 1 were more likely to have received local treatment for BM, especially whole-brain irradiation, compared to those in Cohort 2. There was no difference in survival between patients in Cohort 1 and those in Cohort 2. Eighth, the last patient was enrolled 10 years ago in our study; however, the analyses were done later in a retrospective study. We used the data from the HOT 1701 study that enrolled patients between January 2008 and December 2014. Our analyses and study still hold significance because, to the best of our knowledge, this is the first study to develop a prognostic model for Japanese patients who had BM at diagnosis. The prognostic score, specifically developed for the Japanese patients, may help determine the treatment plan of the affected patients in the future after the score is validated in external cohorts. Ninth, in this study, we investigated lung cancer cases with BM at initial diagnosis. Unfortunately, we were unable to evaluate whether there were differences between those who developed BM as a recurrence/progression of the disease and those who had metastatic disease at initial diagnosis. Finally, inter-institutional variability in clinical management might have existed in our study. However, the literature demonstrates that institutional practice patterns are highly influenced by local practice and likely stem from the beliefs/preferences of local leaders, especially with respect to the adoption of new technologies.^31^

In conclusion, the univariate and multivariate analyses in our study led to the identification of 6 independent prognostic factors: age ≥65 years, poor PS, T factor, absence of driver gene mutations, presence of ECM, and number of BM. Stratification of patients according to risk based on these prognostic factors may help determine the prognosis of patients at initial diagnosis, leading to appropriate treatment.

## Supplementary Material

vdae168_suppl_Supplementary_Material

## Data Availability

The datasets used and/or analyzed during the current study are available from the corresponding author on reasonable request.
