# Supplementary material for: Non-small cell lung cancer with synchronous brain metastases: Identification of prognostic factors in a retrospective multicenter study (HOT 1701)
Source: Neurooncol Adv. 2024 Oct 5;6(1):vdae168. doi: 10.1093/noajnl/vdae168 (PMC11558066; doi:10.1093/noajnl/vdae168)
Supplement: vdae168_suppl_Supplementary_Material [file vdae168_suppl_Supplementary_Material.docx]

**Non-small cell lung cancer with synchronous brain metastases: Identification of prognostic factors in a retrospective multicenter study (HOT 1701)**

**Supplementary Material**


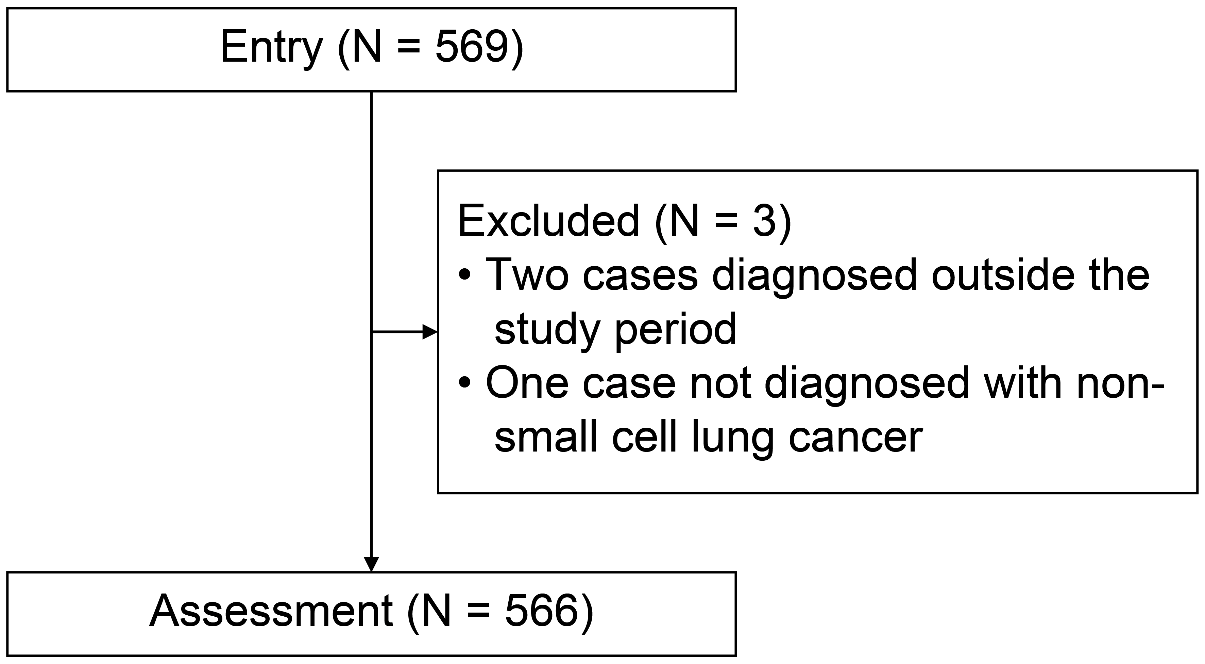


**Supplementary Figure 1.** Flowchart for patient enrolment in the study


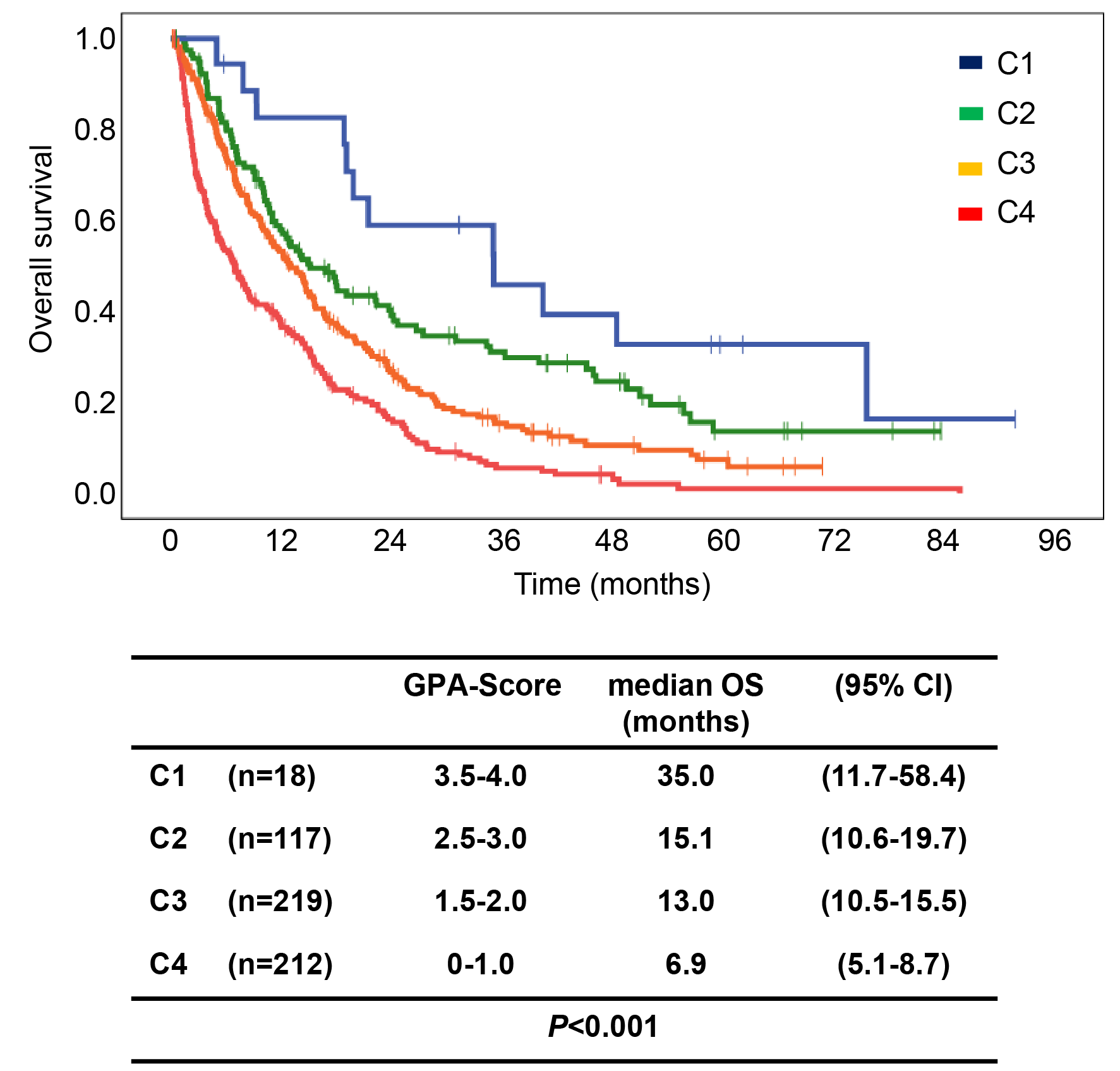


**Supplementary Figure 2.** Graded prognostic assessment (GPA) score analyzed using the data of patients enrolled in our study.

**Supplementary Table 1. Staging systems used for analysis**

| TX | Primary tumor cannot be assessed, or tumor proven by the presence of malignant cells in sputum or bronchial washings but not visualized by imaging or bronchoscopy |
| --- | --- |
| T1 | Tumor < 3 cm in the greatest dimension, surrounded by lung or visceral pleura, without bronchoscopic evidence of invasion more proximal than the lobar bronchus (i.e., not in the main bronchus) |
| T2 | Tumor > 3 cm but < 7 cm or tumor with any of the following features (T2 tumors with these features are classified T2a if <5 cm):  Involves main bronchus, >2 cm distal to the carina  Invades visceral pleura  Associated with atelectasis or obstructive pneumonitis that extends to the hilar region but does not involve the entire lung |
| T3 | Tumor > 7 cm or one that directly invades any of the following:  Chest wall (including superior sulcus tumors), diaphragm, phrenic nerve, mediastinal pleura, parietal pericardium  Tumor in the main bronchus < 2 cm distal to the carina but without involvement of the carina  Associated atelectasis or obstructive pneumonitis of the entire lung  Separate tumor nodule(s) in the same lobe |
| T4 | Tumor of any size that invades any of the following:  Mediastinum, heart, great vessels, trachea, recurrent laryngeal nerve, esophagus, vertebral body, carina  Separate tumor nodule(s) in a different ipsilateral lobe |
| N: Nodes | |
| Nx | Regional lymph nodes cannot be assessed |
| N0 | No regional lymph node metastasis |
| N1 | Metastasis in ipsilateral peribronchial and/or ipsilateral hilar lymph nodes and intrapulmonary nodes, including involvement by direct extension |
| N2 | Metastasis in ipsilateral mediastinal and/or subcarinal lymph node(s) |
| N3 | Metastasis in contralateral mediastinal, contralateral hilar, ipsilateral or contralateral scalene, or supraclavicular lymph node(s) |

**Supplementary Table 2. Clinicopathological features of adenocarcinoma and squamous cell carcinoma in lung cancer with brain metastasis**

|  |  | Adenocarcinoma | Squamous cell carcinoma | *P* value |
| --- | --- | --- | --- | --- |
|  |  | (N = 431) | (N = 67) |  |
| Sex | Male | 250 | 50 | 0.01 |
|  | Female | 181 | 17 |  |
| Age (years) | <65 | 194 | 19 | 0.01 |
|  | ≥65 | 237 | 48 |  |
| Smoking habit | Never | 133 | 10 | 0.007 |
|  | Other | 292 | 56 |  |
| ECOG PS | 0–1 | 293 | 44 | 0.707 |
|  | 2–4 | 138 | 23 |  |
| T factor | T1–2 | 216 | 25 | 0.052 |
|  | T3–4 | 210 | 41 |  |
| N factor | N0 | 79 | 8 | 0.198 |
|  | N1–3 | 351 | 59 |  |
| Extracranial metastases | Absent | 105 | 29 | 0.001 |
|  | Present | 326 | 38 |  |
| Driver mutation | No | 262 | 64 | <0.001 |
| (*EGFR, ALK*) | Yes | 169 | 3 |  |
| Number of BM | 1–3 | 226 | 51 | <0.001 |
|  | ≥4 | 205 | 16 |  |
| Symptoms of BM | Absent | 260 | 46 | 0.2 |
|  | Present | 170 | 21 |  |
| Local treatment for BM | No | 121 | 20 | 0.764 |
|  | Yes | 310 | 47 |  |
| Whole-brain radiotherapy | No | 234 | 36 | 0.932 |
|  | Yes | 197 | 31 |  |
| SRT | No | 299 | 47 | 0.898 |
|  | Yes | 132 | 20 |  |
| Surgery | No | 387 | 63 | 0.274 |
|  | Yes | 44 | 4 |  |
| Chemotherapy | No | 82 | 19 | 0.077 |
|  | Yes | 349 | 48 |  |

ECOG, Eastern Cooperative Oncology Group; PS, performance status; EGFR, epidermal growth factor receptor; ALK, anaplastic lymphoma kinase; BM, brain metastases; SRT, stereotactic radiosurgery or stereotactic radiotherapy

**Supplementary Table 3. Clinicopathological features of patients enrolled in 2008–2011 and those in 2012–2014**

|  |  | Cohort 1 | Cohort 2 | P value |
| --- | --- | --- | --- | --- |
|  |  | (2008–2011) | (2012–2014) |  |
| Sex | Male | 127 | 223 | 0.771 |
|  | Female | 81 | 135 |  |
| Age (years) | <65 | 99 | 147 | 0.131 |
|  | ≥65 | 109 | 211 |  |
| Smoking habit | Never | 66 | 87 | 0.047 |
|  | Other | 138 | 267 |  |
| Staging | Stage IV | 200 | 339 | 0.432 |
|  | Recurrence | 8 | 19 |  |
| ECOG PS | 0–1 | 140 | 234 | 0.638 |
|  | 2–4 | 68 | 124 |  |
| T factor | T1–2 | 98 | 175 | 0.71 |
|  | T3–4 | 107 | 179 |  |
| N factor | N0 | 36 | 62 | 0.986 |
|  | N1–3 | 172 | 295 |  |
| Extracranial metastases | Absent | 60 | 96 | 0.602 |
|  | Present | 148 | 262 |  |
| Driver mutation | No | 153 | 237 | 0.068 |
| (*EGFR, ALK*) | Yes | 55 | 121 |  |
| Number of BM | 1–3 | 117 | 200 | 0.929 |
|  | ≥4 | 91 | 158 |  |
| Symptoms of BM | Absent | 116 | 215 | 0.3 |
|  | Present | 92 | 142 |  |
| Local treatment for BM | No | 40 | 111 | 0.002 |
|  | Yes | 168 | 247 |  |
| Whole-brain radiotherapy | No | 88 | 208 | <0.001 |
|  | Yes | 120 | 150 |  |
| SRT | No | 149 | 246 | 0.466 |
|  | Yes | 59 | 112 |  |
| Surgery | No | 183 | 323 | 0.403 |
|  | Yes | 25 | 35 |  |
| Chemotherapy | No | 48 | 77 | 0.664 |
|  | Yes | 160 | 281 |  |

ECOG, Eastern Cooperative Oncology Group; PS, performance status; EGFR, epidermal growth factor receptor; ALK, anaplastic lymphoma kinase; BM, brain metastases; SRT, stereotactic radiosurgery or stereotactic radiotherapy
